# Supplementary material for: Sporadic Amyotrophic Lateral Sclerosis Skeletal Muscle Transcriptome Analysis: A Comprehensive Examination of Differentially Expressed Genes
Source: Biomolecules. 2024 Mar 20;14(3):377. doi: 10.3390/biom14030377 (PMC10967759; doi:10.3390/biom14030377)
Supplement: Supplementary file 1 [file biomolecules-14-00377-s001.zip › Gasc. E. - ALS Skeletal Muscle. Supplementary Material.pdf]

**Supplementary Table S1.** List of abbreviations.

| Abbreviations | Name |
|---|---|
| ALS | Amyotrophic Lateral Sclerosis |
| sALS | Sporadic Amyotrophic Lateral Sclerosis |
| fALS | Familiar Amyotrophic Lateral Sclerosis |
| SOD1 | Superoxide Dismutase 1 |
| FUS | FUS RNA Binding Protein |
| C9ORF72 | C9orf72-SMCR8 Complex Subunit |
| ATXN2 | Ataxin 2 |
| OPTN | Optineurin |
| VCP | Valosin Containing Protein |
| PFN1 | Profilin 1 |
| MATR3 | Matrin 3 |
| SETX | Senataxin |
| UBQLN2 | Ubiquilin 2 |
| LGALSL | Galectin Like |
| FIG4 | FIG4 Phosphoinositide 5-Phosphatase |
| ALS2 | Alsin Rho Guanine Nucleotide Exchange Factor ALS2 |
| GEO | Gene Expression Omnibus |
| GSE | Gene Expression Series |
| DEG | Differentially Expressed Genes |
| GO | Gene Ontology |
| BP | Biological Process |
| CC | Cellular Component |
| MF | Molecular Function |
| PPI | Protein-protein Interaction |
| MCC | Maximum clique centrality |
| EIF4A1 | Eukaryotic Translation Initiation Factor 4A1 |
| CCT2 | CCT2: Chaperonin Containing TCP1 Subunit 2 |
| ETF1 | Eukaryotic Translation Termination Factor 1 |
| PABPC1 | Poly(A) Binding Protein Cytoplasmic 1 |
| HNRNPR | Heterogeneous Nuclear Ribonucleoprotein R |
| EIF3A | Eukaryotic Translation Initiation Factor 3 Subunit A |
| EEF2 | Eukaryotic Translation Elongation Factor 2 |
| HNRNPA1 | Heterogeneous Nuclear Ribonucleoprotein A1 |
| RPLP0 | Ribosomal Protein Lateral Stalk Subunit P0 |
| EEF1A1 | Eukaryotic Translation Elongation Factor 1 Alpha 1 |
| RAN | RAN, Member RAS Oncogene Family |
| RPL12 | Ribosomal Protein L12 |
| CCT6A | Chaperonin Containing TCP1 Subunit 6A |
| RPL15 | Ribosomal Protein L15 |
| CCT3 | Chaperonin Containing TCP1 Subunit 3 |
| COX5B | Cytochrome C Oxidase Subunit 5B |
| COX6A2 | Cytochrome C Oxidase Subunit 6A2 |
| NDUFA4 | NDUFA4 Mitochondrial Complex Associated |
| COX6C | Cytochrome C Oxidase Subunit 6C |

| | |
|---|---|
| NDUFB4 | NADH:Ubiquinone Oxidoreductase Subunit B4 |
| ATP5MC1 | ATP Synthase Membrane Subunit C Locus 1 |
| COX8A | Cytochrome C Oxidase Subunit 8A |
| NDUFA3 | NADH:Ubiquinone Oxidoreductase Subunit A3 |
| ATP5PF | ATP Synthase Peripheral Stalk Subunit F6 |
| COX7A1 | Cytochrome C Oxidase Subunit 7A1 |
| COX5A | Cytochrome C Oxidase Subunit 5A |
| NDUFA6 | NADH:Ubiquinone Oxidoreductase Subunit A6 |

**Supplementary Table S2.** GSE41414 Sample Set Information.

| ID | Group | Site of onset | Gender | Age | Biopsied muscle |
|---|---|---|---|---|---|
| 1 | Control | - | Male | 59 | Deltoid |
| 2 | Control | - | Female | 62 | Deltoid |
| 3 | Control | - | Male | 66 | Deltoid |
| 4 | Control | - | Female | 60 | Deltoid |
| 5 | Control | - | Female | 64 | Deltoid |
| 6 | Control | - | Female | 70 | Deltoid |
| 7 | Control | - | Male | 65 | Deltoid |
| 8 | ALS | Spinal | Male | 73 | Quadriceps |
| 9 | ALS | Spinal | Female | 72 | Quadriceps |

| | | | | | |
|---|---|---|---|---|---|
| 10 | ALS | Spinal | Female | 59 | Deltoid |
| 11 | ALS | Bulbar | Male | 54 | Deltoid |
| 12 | ALS | Spinal | Male | 72 | Deltoid |
| 13 | ALS | Spinal | Female | 55 | Deltoid |
| 14 | ALS | Bulbar | Male | 64 | Quadriceps |
